# Youth Metacognitive Therapy (YoMeta): protocol for a single-blind randomised feasibility trial of a transdiagnostic intervention versus treatment as usual in 11–16-year-olds with common mental health problems

**DOI:** 10.1186/s40814-022-01162-5

**Published:** 2022-09-12

**Authors:** Adrian Wells, Karin Carter, Mark Hann, Gemma Shields, Paul Wallis, Beth Cooper, Lora Capobianco

**Affiliations:** 1 School of Psychological Sciences, Faculty of Biology, Medicine and Health, The University of Manchester, Manchester, UK; 2 Research and Innovation, Greater Manchester Mental Health NHS Foundation Trust, Manchester, UK; 3 Division of Population Health, Health Services Research, and Primary Care, Biostatistics Group, Faculty of Biology, Medicine, and Health, The University of Manchester, Manchester, UK; 4 Division of Population Health, Health Services Research, and Primary Care, Manchester Centre for Health Economics, Faculty of Biology, Medicine, and Health, The University of Manchester, Manchester, UK; 5 Child and Adolescent Mental Health Services, Manchester University NHS Foundation Trust, Manchester, UK

**Keywords:** Metacognitive therapy, Mental health, Anxiety, Depression, Children, Adolescents, CAMHS

## Abstract

**Background:**

Mental health disorders in children and young people (CYP) are increasing but the provision of current evidence-based treatment for common mental health problems is limited. Treatment effects vary widely with no clear superiority of a single treatment approach. Further evaluation of contemporary and effective treatments in CYP is needed. Metacognitive therapy (MCT) has shown enhanced efficacy over ‘gold standard’ approaches in adult mental health, but so far has not been evaluated in a randomised trial of CYP. As such, we aim to assess the acceptability and feasibility of group-MCT for CYP with common mental health problems in comparison to usual treatment within Child and Adolescent Mental Health Services (CAMHS).

**Method:**

YoMeta is a multicentre, two-arm, single-blind randomised feasibility trial comparing group-MCT to usual care in CYP with common mental health problems in CAMHS. CYP (target sample *n* = 100) with a common mental health problem will be recruited across at least three CAMHS services in the UK. Participants in the intervention arm will receive up to eight sessions of group-MCT delivered by a CAMHS mental health practitioner. The control arm will receive usual care in CAMHS which includes individual or group-based therapy. Feasibility will be assessed by the success of recruitment, retention, and data quality. Acceptability of the intervention will be assessed by the number of sessions attended and through qualitative interviews aimed at exploring CYP acceptability and understanding of the intervention. Symptoms of psychological distress will be assessed using the Revised Children Anxiety and Depression Scale (RCADS) at 20 weeks. We will also assess psychological well-being, symptoms of depression, metacognitive beliefs, quality of life, and measures to support economic evaluation (health status and health and social care use). Qualitative interviews will be conducted to understand practitioner’s views on training and delivery of group-MCT.

**Discussion:**

The trial is designed to evaluate the acceptability and feasibility of group-MCT for CYP with common mental health problems. Group-MCT may aid in improving access to treatment, reduce waiting times, and improve outcomes for CYP with common mental health disorders. The study will provide important information and data to evaluate future research potential and confirm sample size estimation for a definitive large-scale RCT to test the effectiveness and cost-effectiveness of group-MCT in CYP.

**Trial registration:**

NCT05260060; ISCTRN18335255

**Supplementary Information:**

The online version contains supplementary material available at 10.1186/s40814-022-01162-5.

## Introduction

Mental health problems in children and young people (CYP) are rising. In 2021, the National Health Service (NHS) reported that 17.4% or one in six children aged 6 to 16 years had a probable mental health disorder, an increase from 12.8% in the 2017 NHS report [1, 2]. Mental health problems in young people are associated with poorer outcomes later in life, including further mental health problems, decreased lifetime earnings, increased drug and alcohol use, greater criminal activity, and increased healthcare costs [3–6]. An increasing prevalence of mental health disorders is placing pressure on Child and Adolescent Mental Health Services (CAMHS) in the UK. The annual report from the Children’s Commissioner on Mental Health Services estimates that only 32% of CYP with a probable mental health disorder are able to access treatment [7]. Despite a general downward trend in waiting time to treatment since 2015, average waiting times to access treatment exceed the UK government’s 4-week wait target, with average waiting times varying by area from 8 days to 82 days [8]. However, the NHS proxy for ‘entering treatment’ is to receive two contacts. On average, services with the largest decreases in waiting times saw increases in numbers of CYP waiting without two contacts, indicating that a large proportion of children are being placed on waiting lists rather than receiving ‘treatment’ [7].

Current interventions for mental health disorders in CYP primarily use cognitive behavioural therapy (CBT) delivered in individual, group, or family/parent formats. Cochrane reviews have highlighted that CBT can be effective when compared to a no-intervention control for CYP with anxiety disorders [8–10]. In a review of 87 randomised controlled trials (RCTs) evaluating treatment of anxiety disorders [9], most studies favoured CBT over waitlist control on diagnostic remission and anxiety symptom scores. However, there were no differences amongst the small number of studies reporting follow-up data. When comparing CBT with active control conditions, no significant differences were found in diagnostic remission or improved anxiety symptoms. Eight studies compared CBT with treatment as usual, where differences were found to be non-significant. The studies reviewed excluded young people diagnosed with PTSD or OCD. However, specific reviews show that behaviour therapy or CBT is more effective than waitlist or pill placebo in treating OCD, but superiority against psychological placebo was not found in one study [11]. In the matter of PTSD, a range of different therapies (e.g. CBT, EMDR, psychodynamic, counselling) appeared to improve symptoms compared to control conditions within a month of completing treatment, but evidence of superiority of a particular type of treatment is insufficient [12]. Outcomes in depression treatment have shown the effects of CBT to be moderate compared with attention placebo (*g* = .54), and waitlist (*g* = .70) [13]. Earlier reviews did not find significant differences between individual or combination treatments (antidepressant + CBT) [14]. However, more extensive reviews show that fluoxetine in combination with CBT may be more effective than CBT alone or psychodynamic therapy alone, but not more effective than fluoxetine alone [15]. Fluoxetine plus CBT or fluoxetine alone was superior to pill placebo or psychological controls but there is evidence that specific antidepressants (venlafaxine) are associated with adverse events [15].

In summary, a range of treatments appear effective in treating child and adolescent anxiety disorders but with limited evidence of the superiority of any treatment type. In depression, fluoxetine plus CBT is more effective than CBT alone but not more effective than fluoxetine alone. There is a wide range of treatment effect sizes, a limited amount of follow-up data and samples drawn from sources such as schools rather than mental health settings, potentially impacting on the reliability and generalisability of findings.

There are multiple challenges in providing sustainable and effective child and adolescent treatment for common mental health problems. There is a need to deal with the increasing demand for services, to provide greater access, and to offer new and more effective treatment options. A therapeutic approach that may help to meet these objectives that is proving to be effective in adult mental health is metacognitive therapy (MCT: [16]). MCT offers benefits over approaches such as CBT since it aims to modify transdiagnostic processes thought to be central to the maintenance of most common mental health problems. The approach can therefore offer a standard set of techniques for dealing with a diverse set of presentations at the same time, which is useful for multimorbidities at the individual level and for combining a range of patients in cost-effective treatment groups.

MCT differs from CBT as it targets the regulation of common processes (i.e. worry and repetitive negative thinking) across psychological disorders, hypothesised to be central to the development and maintenance of most common mental health symptoms.

MCT has shown promising results in small uncontrolled studies of CYP across a range of disorders including anxiety [17], post-traumatic stress disorder [18], and obsessive-compulsive disorder [19]. For example, a within-subjects trial of group-MCT for children with generalised anxiety disorder (aged 7–13) showed that treatment was associated with large effect sizes at post-treatment on the Revised Children Anxiety and Depression Scale (RCADS) [17], with 70% of children classified as improved at post-treatment. These positive results were maintained at follow-up.

While the results of MCT in CYP are promising, most studies have been completed in disorder-specific contexts and all studies are uncontrolled. As such, we do not know about the acceptability and feasibility of conducting a randomised trial of MCT in a transdiagnostic group format in this population.

The current study aims to address this important gap in the literature by evaluating the acceptability and feasibility of MCT in a mixed mental health group (i.e. children with a range of anxiety, stress, and mood disorder symptoms) in comparison to treatment as usual (TAU).

## Methods

### Design

**Fig. 1:**
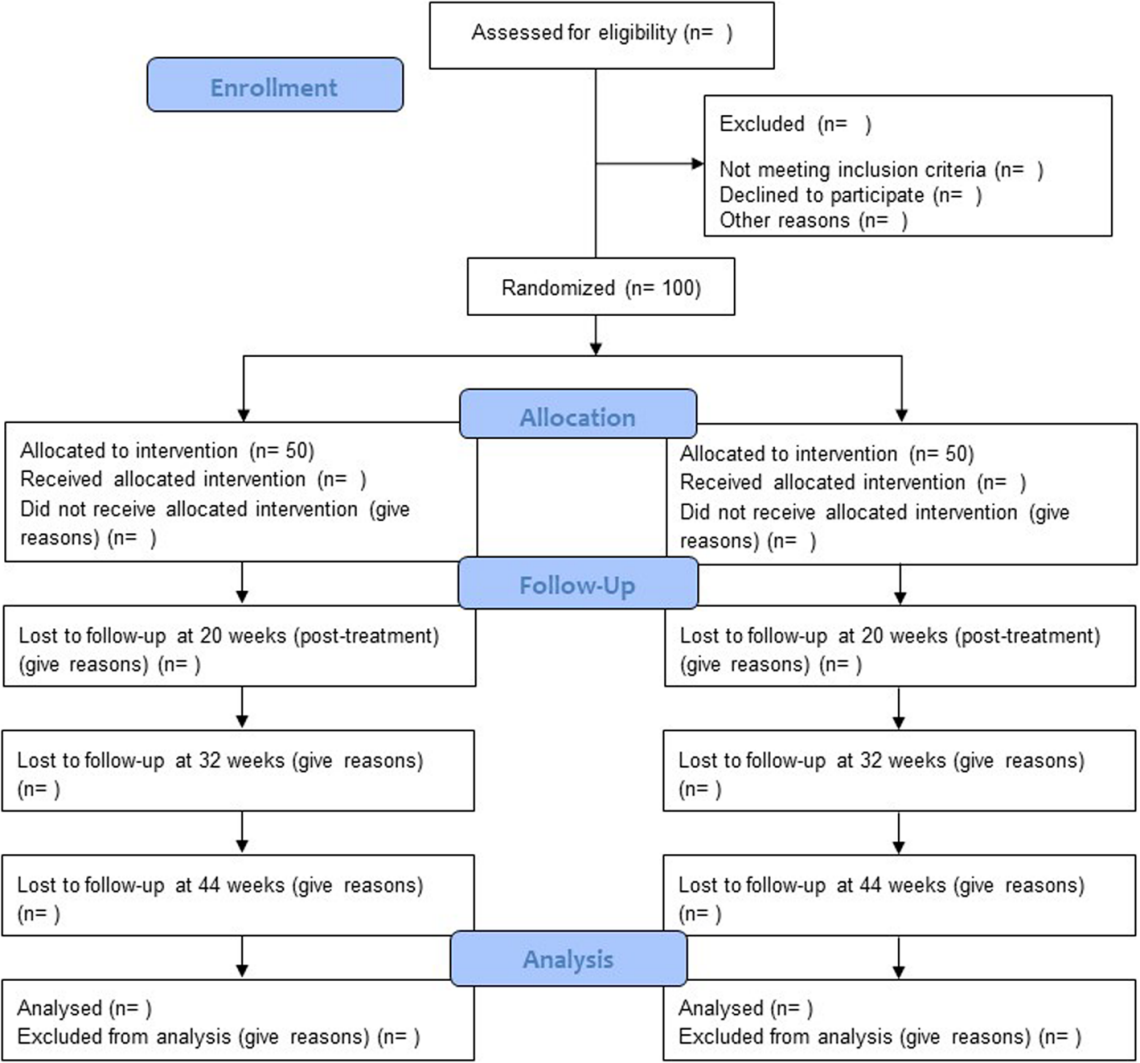
An overview of the trial design according to CONSORT guidelines

**Fig. 2:**
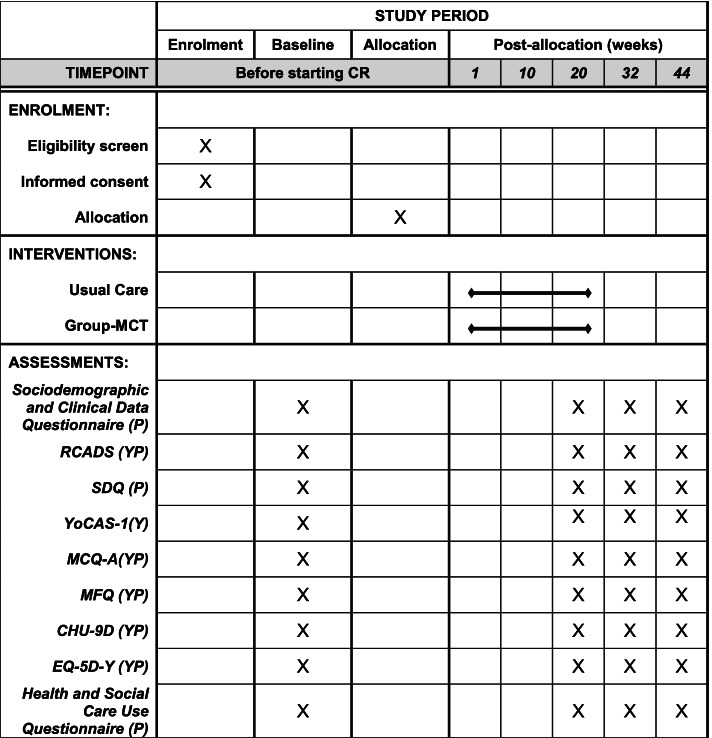
Schedule of enrolment, interventions, and assessments. Group-MCT, group-based metacognitive therapy; RCADS, Revised Children Anxiety and Depression Scale-Short version; SDQ, Strength and Difficulties Questionnaire; YoCAS-1, Youth Cognitive Attentional Syndrome-1; MCQ-A, Metacognition Questionnaire-Adolescent version; MFQ, Mood & Feelings Questionnaire; CHU-9D, Child Health Utility-9D; EQ-5D-Y; SUI, Health and Social Care Service-Use Interview; P, parent rated; YP, young person rated

Youth Metacognitive Therapy (YoMeta) is a single-blind, randomised feasibility study with 20 weeks, 32 weeks, and 44 weeks of follow-up, comparing group-based MCT (intervention) versus usual treatment (control). Qualitative evaluations will be embedded within the trial. Preliminary economic data will be obtained to inform the optimum way of evaluating cost-effectiveness. Figure 1 shows an overview of the trial design according to CONSORT guidelines [20, 21]. The recommendations for the Interventional Trials 2013 (SPIRIT) Checklist [22, 23] are included in Additional file 1. Figure 2 outlines the schedule of recruitment, interventions, and assessment.

### Trial population

The trial population are children and adolescents referred to CAMHS at three Greater Manchester services at Manchester University Foundation NHS Trust.

### Eligibility criteria

In order to be eligible to take part in the study, participants will meet the following inclusion and exclusion criteria:

#### Inclusion criteria

1. Aged between 11 and 16 years
2. Consent given
3. Native fluency in the English language
4. Seeking treatment for emotional disorder symptoms (i.e. generalised anxiety disorder, panic disorder, agoraphobia, post-traumatic stress disorder, obsessive-compulsive disorder, social anxiety, and/or depression)
5. Medication for mental health problems is permitted but participants must be stabilised for 6 weeks

Participants will be excluded if they meet one or more of the following:

1. Presence of significant risk or safeguarding concerns
2. Head injury/organic impairment
3. Referred for autism spectrum disorder, attention deficit-hyperactivity disorder
4. Eating disorder

Only patients with a formal diagnosis or under assessment for one of the exclusion criteria will be excluded from the study.

### Recruitment and allocation

Clinicians in the CAMHS services will assess patients at their usual assessment appointment. If a patient meets the study eligibility criteria, they will be provided with study information and their contact details will be provided to the study team. Researchers will contact the parents/caregivers of eligible and interested patients to provide further details on the study, answer any questions, arrange to obtain informed consent, and collect baseline data. Once baseline data has been collected, participants will be allocated to a study arm. An unblind member of the research team will contact the Centre for Biostatistics at the University of Manchester via a telephone link to randomise patients The first six participants will be allocated 1:1, at random, in a single block of size 6. Thereafter, allocation will be by minimisation based on age (11–13; 14–16), sex, primary presenting problem (anxiety, depression, or both), and study site and will be conducted by an independent statistician. We will use a weighting of 4-to-1 so that there is an 80% chance that the next participant will be allocated to the study arm that minimises the imbalance between the four variables listed above. A member of the research team not blinded to allocation will then contact parents/caregivers to inform them of the allocation within 2 days. Blinding of allocation will be maintained for research assistants until all outcome measures for all subjects have been collected. The Trial Executive Committee (TEC) will regularly monitor unblinding’s and corrective action will be implemented if needed.

## Trial conditions

### Group metacognitive therapy (group-MCT)

Group-MCT will include 8 weekly sessions of group-based MCT. Sessions will last approximately 90 min and be guided by a treatment manual to maximise treatment adherence and fidelity. Groups may range from a minimum of 3 participants to a maximum of 10 participants, with the same therapists at each site delivering all eight sessions.

Group-MCT aims to help participants develop knowledge that can facilitate control of worry, repetitive negative thinking, and attention and to modify the metacognitive beliefs that maintain unhelpful thinking patterns [16].

Eight main treatment techniques will be used across sessions: (1) formulation, (2) socialisation, (3) the Spatial Attentional Control Exercise (SpACE), (4) detached mindfulness, (5) worry and rumination postponement, (6) modifying metacognitive beliefs about worry and rumination, (7) a ‘helpful behaviours prescription’, and (8) individual treatment summaries. Sessions include group discussions, experiential learning, and homework tasks that participants are asked to complete between sessions.

### Treatment as usual (TAU)

TAU will consist of routine care delivered within CAMHS. Therapies delivered include but are not limited to cognitive behavioural-based therapies (CBT), behavioural activation, exposure therapy, Eye Movement Desensitization and Reprocessing (EMDR), and family therapy delivered on a one-to-one or group basis. Participants normally receive up to 12 (1-h) sessions of TAU.

## Staff training and supervision

Two mental healthcare professionals per site will be trained to deliver group-MCT. Staff will receive 2.5 days of training in group-MCT and deliver a pilot group. Staff will receive routine supervision in group-MCT throughout the trial. Group-MCT sessions will be audio recorded and audio tapes will be reviewed in supervision to ensure treatment adherence and fidelity. Any staff trained in group-MCT who also deliver TAU to a trial patient will audio record their TAU sessions, of which a random sample will be assessed for cross-contamination and treatment drift.

## Data collection

There will be four assessment time points: baseline, 20 weeks post-randomisation, 32 weeks post-randomisation, and 44 weeks post-randomisation (see Figs. 1 and 2). In order to aid the return of follow-up measures, participants will be offered a range of options for completing measures including returning via post, telephone, or face-to-face (at NHS sites or participants’ home). Participants’ time involvement in the study is 44 weeks. Participants will be offered compensation for completing assessments; each time an assessment is completed and returned, they will be reimbursed with a £10 shopping voucher.

## Criteria for discontinuation

Participants have the right to withdraw from the study at any time. Discontinuation will not impact on the patient’s healthcare or their ability to take part in future research. Participants who withdraw will continue to receive usual care. Participants can also be withdrawn at the request of the chief investigator, but this would only happen in the event that a participant’s life or long-term health or welfare was at risk from continued participation in the study.

## Outcomes

The primary outcomes for the study are feasibility and acceptability outcomes. Feasibility will be assessed using referral rates, recruitment and retention rates, participant attendance at sessions, their follow-up and questionnaire response rates, and willingness to receive either treatment. Qualitative interviews will also be used to assess patients’ views of the acceptability of the intervention.

## Measures

### Revised Child Anxiety and Depression Scale-Short Version (RCADS-25 [24])

RCADS is a 25-item self-report measure of anxiety (15 items) and depression (10 items) suitable for children aged 8 to 18 [24, 25]. Respondents indicate how often each item occurs with items rated on a 4-point (0 = ‘Never’ to 3 = ‘always’) Likert scale. Scores for the total anxiety scale range from 0 to 45 and from 0 to 30 for the total depression scale; total measure scores range from 0 to 75 with higher scores indicating the level of clinical severity. The subscales have acceptable reliability (total anxiety Cronbach’s alpha = 0.91, total depression Cronbach’s alpha = 0.80) in clinical samples and are frequently delivered in CAMHS [25]. The RCADS is provisionally intended to be the primary outcome in a subsequent definitive trial.

### Strength and Difficulties Questionnaire (SDQ [26])

The Strength and Difficulties Questionnaire is a brief behavioural measure completed by the caregiver or teacher for children aged 2 to 17, across five subscales: emotion, hyperactivity, conduct, peer relations, and pro-social behaviour. Each subscale contains five items, and each item is scored ‘Not True’, ‘Somewhat True’, or ‘Completely True’. Scores for the subscales range from 0 to 10, with total difficulties score (0–40) generated by summing together the scores from all subscales except pro-social. Higher scores on the pro-social scale reflect strengths whereas higher scores across other subscales reflect difficulties [26]. The SDQ subscales have satisfactory internal consistency (mean Cronbach’s alpha = 0.70) and test-retest reliability ranging from 0.75 to 0.91 [27].

### Metacognition Questionnaire-Adolescent version (MCQ-A [28])

The MCQ-A assesses metacognitive beliefs (beliefs about thinking) across five subscales: uncontrollability and dangerousness of worry, need to control thoughts, cognitive self-consciousness, positive beliefs about worry, and cognitive confidence. The 30 items are scored on a Likert scale from 1 (do not agree) to 4 (agree very much) with total scores ranging from 30 to 120 and 6 to 24 for each subscale [28]. The internal consistency of the MCQ-A across total scores and most subscales has been supported with adequate to excellent Cronbach alphas (0.76–0.92) [29].

### Mood & Feelings Questionnaire (MFQ [30])

The MFQ measures depressive symptoms (feelings or behaviours) in 6–19-year-olds over 33 items regarding how the individual has been feeling over the past 2 weeks. Items are scored from 0 (not true) to 2 (true) with total scores ranging from 0 to 66, higher scores indicating more severe depressive symptoms [30]. The MFQ has demonstrated excellent internal consistency (Cronbach’s alphas = 0.91 to 0.93) [31].

### Youth Cognitive Attentional Syndrome-1 (YoCAS-1 [32])

The YoCAS-1 is a 7-item measure that assesses features of the cognitive attentional syndrome (i.e. extended negative thinking/coping styles) [16]. The YoCAS-1 measures positive and negative metacognitive beliefs, maladaptive coping strategies, and amount of time spent worrying and dwelling on negative thoughts. Items are rated based on the past 7 days on an 11-point response scale ranging from 0 (none of the time/not at all true) to 100 (all of the time/completely certain this is true) in increments of 10. The YoCAS-1 was adapted from the CAS-1 [16]; modifications were guided by feedback from service users to ensure that the language is appropriate for use with children and adolescents.

### Child Health Utility-9D (CHU-9D [33])

The CHU-9D measures paediatric quality of life using 9 dimensions (including worried, sad, pain, tired, annoyed, schoolwork/homework, sleep, daily routine, and ability to join in activities) suitable for 7–17-year-olds. Each item has 5 levels ranging from no problems to inability to do the item [33]. The CHU-9D has adequate internal consistency (Cronbach’s alpha = 0.79) and test-retest reliability (interclass correlation coefficients = 0.75) [34].

### EQ-5D-Y [35]

The EQ-5D-Y measures general health status using five dimensions (mobility, looking after myself, doing usual activities, having pain or discomfort, and feeling worried, sad or unhappy). Each dimension has three levels indicating the degree of health impact on the activity (none, some, a lot) with individuals required to give a response relating to health on the day of completion [34]. The EQ-5D-Y has shown to be a valid and reliable measure of health-related quality of life in children, with fair to strong test-retest reliability (70–99%) [36].

### Demographic information questionnaire

The demographic questionnaire will collect variables including child’s age, sex, school, ethnicity, medication, socioeconomic status, and parental occupational status.

### Health and Social Care Service-Use Questionnaire (SUQ)

The SUQ will include questions about whether the child has used any primary, secondary, or community-based health and social care and how often they used the service in the last 16 weeks (baseline study visit) or since the last assessment (follow-up study visits). The SUQ will be developed from existing child-relevant SUQs held by the co-applicants and through discussion with the PPI representative, parent advisory group, and clinical members of the study team. Parents/primary caregivers will complete this.

## Qualitative evaluation

Qualitative interviews will be conducted to evaluate patients’ and clinician’s perspectives of MCT. We will conduct semi-structured interviews with around 10–20 patients and 4–6 clinicians trained in MCT.

Patient interviews will evaluate which aspects of group-MCT they enjoyed/learned from most as well as the aspects that they found most difficult to understand/engage in. We will also assess homework compliance and ways in which this could be improved.

Qualitative interviews with clinicians will evaluate experiences of being trained in MCT and delivering MCT, which aspects of MCT were challenging/easy to deliver, and experiences of delivering MCT to a mixed disorder group.

## Sample size calculation

Feasibility trials are not powered to provide a definitive effectiveness analysis. The sample size is, therefore, based on having sufficient numbers of patients to evaluate the acceptability/feasibility of group-MCT (as measured by patient acceptability and adherence ratings, recruitment, and retention rates) and also to obtain a sufficiently precise provisional estimate of the ‘promise’ of the intervention (with 95% C.I.) for powering a future definitive RCT. To this end, we will recruit 50 patients per arm (total *N* = 100) which, for example, would allow estimation of the retention rate to within 10 absolute percentage points with 95% confidence. The total sample is also more than adequate for estimation of the variability in outcome measures, such as the RCADS-25, for which samples of 40 are generally sufficient [37, 38].

## Analyses

### Quantitative analyses

As the study is a feasibility study, we will not be carrying out hypothesis testing to determine if the intervention is effective. Data analysis will follow an intention-to-treat (ITT) protocol and will be used to inform power calculations for a definitive trial in addition to other published sources.

We will calculate and present in a CONSORT flow chart: the number of CYP approached to participate, the number consenting to participate in the study, the number completing the baseline assessment and subsequently allocated to each trial arm, the number of therapy sessions attended by CYP receiving the MCT intervention and the proportion completing the treatment (defined as attending a minimum of four out of eight sessions), and the number completing each of the follow-up assessments in each trial arm. Reasons for drop-out will be recorded where possible and used to help determine the acceptability of the MCT intervention, along with the attrition rates themselves. We will also assess rates of missing data on individual questionnaires (and which elements in particular) and whether any display floor and/or ceiling effects. The acceptability of minimisation will be informed by examining how balanced treatment allocation is across the four factors on which participants are allocated.

We will summarise, as appropriate (e.g. mean/standard deviation; median/inter-quartile range; proportion/95% confidence interval; data range) data for all potential ‘clinical’ outcome measures, overall and by group. The SD of the (potential) primary outcome for a full trial (RCADS-25), along with the estimated attrition rate, will be used to help inform the sample size calculation for the definitive RCT. As MCT is undertaken as groups, we will attempt to estimate the intra-group correlation coefficient in the RCADS-25, as this may also be used to inform the calculation if appropriate. We will also report confidence intervals for the trial arm coefficient from appropriate regression analyses by way of investigating the ‘promise’ of the intervention. A Statistical Analysis Plan will be produced by the study statistician prior to the examination of outcome data.

### Qualitative analyses

All qualitative interviews will be transcribed verbatim. Analysis will be conducted blind to trial outcomes to avoid biased interpretation of findings. Framework analysis [39] will be used, allowing for both inductive and deductive coding. Deductive coding will be informed by Normalisation Process Theory (NPT) [40].

### Economic analyses

Exploratory analysis will be conducted to inform an economic evaluation integrated within a definitive trial, including an analysis of the range of health and social care services used and associated costs; the ability of the quality-adjusted life-year (QALY) to discriminate between groups based on changes in clinical outcomes; and factors likely to influence the incremental cost per QALY. Two generic preference-based measures are being collected to inform health benefit: the CHU-9D and the EQ-5D-Y. Data will be compared to assess whether one measure is more feasible and preferred for a larger scale trial (e.g. through a comparison of missingness). The CHU-9D will be the primary measure as UK preference weights are available [33]. The impact of the EQ-5D-Y will be tested in a sensitivity analysis. Costs will be estimated from the SUQ data collected, using national unit costs [41, 42]. If the data are sufficient, preliminary cost-effectiveness will be conducted, with a time horizon of 6 months.

#### Trial management and oversight arrangement

The trial is managed by a trial executive committee which consists of the chief investigator, co-investigators, the core project team, and other relevant parties that will meet quarterly. The management team will review study conduct including monitoring study progress, adherence to the protocol, and participant safeguarding. A programme management group comprising the chief investigator and core project team will meet weekly to oversee the day-to-day management of the programme. There is also a service user advisory group which will meet at least every 6 months and provide advice and feedback on a range of trial-related activities, e.g. reviewing study documents. The end of study will be reported to the Research Ethics Committee (REC) within the required timeframe if the study is terminated prematurely. Investigators will inform patients of any premature termination of the study and ensure that the appropriate follow-up is arranged for all involved. Following the end of the study, a summary report of the study will be provided to the REC within the required timeframe.

#### Data management

Participants will be allocated a study identity code for use on all study documents. The research team will create a confidential database of participant identifiable information and study identity code in order to allow the identification of participants enrolled in the study (e.g. for follow-up data collection). Access to study documents will be restricted to authorised persons. Participant consent forms will be filed in the corresponding site file and in participants’ medical notes. Baseline and follow-up data, which is anonymous data, will be stored in locked filing cabinets at GMMH. These data will be entered into an electronic database for analysis purposes by study team members blind to trial arm allocation. All computers are password protected and adhere to the secure storage policies of the NHS trust and University of Manchester.

Data quality checks will be performed on 10% of the data entered electrically from each time point at random by the statistical team. Data quality checks will review database entries with hard-copy questionnaire responses. Any discrepancies will be noted, corrected, and counted to obtain an error rate. Depending on the error rate, further checks will be performed.

#### Safety reporting

Adverse events (AEs) and serious adverse events (SAEs) will be monitored throughout the intervention delivery. Any adverse or serious adverse events identified as likely to be caused by the intervention during the study recruitment or intervention phase will be recorded at the study site using an AE/SAE record form which will be completed by the participants’ health professional delivering treatment. AEs and SAEs will be reported to the research team and reviewed for seriousness and causality by a designated study investigator who is not blind to treatment allocation. Any that are deemed SAEs, and are related to the intervention, will be reported to the ethics committee, the programme executive committee, and the sponsor’s Research and Innovation Manager within 7 days of the event. AEs and SAEs will be reviewed on a quarterly basis at executive committee meetings.

#### Dissemination and publication policy

Study results will be published in peer-reviewed journals and made freely available online where possible. All presentations and publications related to the study will be authorised by the chief investigator and study funder. No investigator may present or attempt to publish data relating to the YoMeta study without prior permission from the chief investigator and the sponsor. The findings will also be presented at national, international, and regional conferences and in public involvement events where the information from this study is relevant.

## Discussion

Mental health disorders are common amongst CYP and have been found to affect 17.4% of individuals with at least 5% experiencing more than one mental health problem [1, 43]. The prevalence of mental health symptoms in CYP is increasing, placing greater demand on child mental health services. The UK government has highlighted the need to improve mental health provision in CYP and reduce the waiting time for treatment of those who need specialist intervention [44, 45].

At present, the effectiveness of current interventions is variable with no clear indication of a predominant psychological treatment approach [9, 10]. As such, therapists are required to be proficient in a range of therapies and to make complex decisions around which problems to deal with first in multiple morbidities or face conceptual difficulties when a specific diagnosis is uncertain. A transdiagnostic intervention, delivered in a group format, could simplify treatment, increase access, improve costs, and decrease waiting times [46–50]. Group-MCT can offer such an approach and has been found to be effective in adult mental health.

MCT is a recent transdiagnostic, evidenced-based psychological therapy based on the metacognitive model of psychology disorder [51–53]. MCT differs from CBT as it targets common processes (i.e., worry, repetitive negative thinking, biased metacognitions) across psychological disorders, hypothesiszed to be central to the development and maintenance of poor mental health. Currently, there are few studies of MCT in CYP and none involving randomised trials. Therefore, the current study will provide evidence of the feasibility and acceptability of running a definitive trial of MCT within CAMHS. The study will provide valuable qualitative and quantitative data to support the design of a future large- scale definitive trial to test the effectiveness of group- MCT for common mental health problems in CYP.

## Supplementary Information

**Additional file 1.** SPIRIT Checklist.

## Data Availability

The datasets generated and/or analysed during the current study are not publicly available due to ongoing data collection but are available from the corresponding author on reasonable request on completion of data collection and data analysis.

## Abbreviations

CAMHS: Child and Adolescent Mental Health Services
CBT: Cognitive behaviour therapy
CYP: Children and young people
MCT: Metacognitive therapy
NHS: National Health Service
TAU: Treatment as usual
QALY: Quality-adjusted life years
